# Experiment for validation of fluid‐structure interaction models and algorithms

**DOI:** 10.1002/cnm.2848

**Published:** 2017-01-27

**Authors:** A. Hessenthaler, N. R. Gaddum, O. Holub, R. Sinkus, O. Röhrle, D. Nordsletten

**Affiliations:** ^1^ Institute of Applied Mechanics (CE) University of Stuttgart Pfaffenwaldring 7 70569 Stuttgart Germany; ^2^ Division of Imaging Sciences and Biomedical Engineering King's College London, 4th Floor, Lambeth Wing St. Thomas Hospital London SE1 7EH UK

**Keywords:** benchmark experiment, fluid‐structure interaction, MRI, validation

## Abstract

In this paper a fluid‐structure interaction (FSI) experiment is presented. The aim of this experiment is to provide a challenging yet easy‐to‐setup FSI test case that addresses the need for rigorous testing of FSI algorithms and modeling frameworks. Steady‐state and periodic steady‐state test cases with constant and periodic inflow were established. Focus of the experiment is on biomedical engineering applications with flow being in the laminar regime with Reynolds numbers 1283 and 651. Flow and solid domains were defined using computer‐aided design (CAD) tools. The experimental design aimed at providing a straightforward boundary condition definition. Material parameters and mechanical response of a moderately viscous Newtonian fluid and a nonlinear incompressible solid were experimentally determined. A comprehensive data set was acquired by using magnetic resonance imaging to record the interaction between the fluid and the solid, quantifying flow and solid motion.

## 1 INTRODUCTION

Mathematical modeling and numerical simulation have become important tools in the investigation of complex multiphysics phenomena^1^ including the interaction between fluids and solids.^2^, ^3^, ^4^, ^5^ In biomedical engineering, fluid‐structure interaction (FSI) modeling is playing an increasingly important role because of the coupling of fluid flow and tissue mechanics vital to many physical phenomena. Use of FSI models for the assessment of medical devices as well as clinical evaluation^6^, ^7^, ^8^ is becoming increasingly common. In silico testing of devices using FSI models can help to expedite and augment preproduction development as well as assist in understanding the implications of an implant and its interaction in the human body. Moreover, in the domain of diagnostics and therapy planning, patient‐specific models of the cardiovascular system^9^, ^10^, ^11^, ^12^ are actively pursued, with the vision of eventually providing clinicians with guidance on treatment.

Practical application of FSI models requires use of both appropriate numerical methods for the problem at hand as well as identification of an appropriate model. Numerical methods for simulating the interaction between fluids and solids are diverse with many variants proposed to address important factors such as large deformations, computational efficiency, ease of implementation naming a few.^13^, ^14^ These methods may rely on varying assumptions on underlying state variables (eg, continuity of pressure between fluid/solid components), discretization strategies, and mechanisms for incorporating coupling. These variations in form make the equivalence of numerical techniques difficult to gauge a priori. Extending beyond the issue of FSI methodology are the practical issues of modeling a physical system. Modeling requires a series of decisions—such as model construction (eg, constitutive laws and material parameters), model boundary conditions, and the degree numerical discretization—that fundamentally influence the accuracy of simulated outcomes. In the biomedical setting, this process is further complicated by the fact that the derivation of geometry and fluid/solid domains often requires imaging data.

Determining the efficacy of FSI modeling approaches, both in numerical methods and modeling approaches, requires verification and validation. In the context of verification, a series of standard benchmark problems have been developed and proposed (see Table 1) to provide a reliable point of comparison on the basis of numerical tests. In this case, fine resolution solutions are calculated for various tests, providing tabulated data to compare with new methods. These examinations are often restricted to 2D because of the constraints on resolution required to obtain accurate solutions with minimal discretization error and to provide more computationally friendly tests for new methods. However, numerical tests for complex 3D FSI problems are far less common, largely because of the increased computational expense. Indeed, without sufficient numerical convergence of the benchmark solution, delineation of a new method's accuracy is virtually impossible to deduce. Beyond verification, there are a number of attempts to provide test cases for validation using physical experiments (see Table 1). These test not only an FSI method's ability to effectively simulate a physical phenomena but also individual modelers on their process for effectively translating a physical problem into a numerical simulation. This provides two sources of error that cannot easily be decoupled, but the sum total can be used to, in some sense, evaluate accuracy. In the 3D setting, validation experiments can avoid potential questions of validity that may be present in numerical benchmarks, being limited only to the accuracy and completeness of the measures they report.

**Table 1:** Selection of popular FSI benchmark test cases (in publication order) with specification of Reynolds number R e and whether the benchmark is based on an experiment

	**FSI benchmark**	**References**	*R* *e*	**Experiment**	**steady/transient**
2D	Flow past elastic cylinder	Ghattas and Li^15^; Wall^16^	50		Steady
2D	Elastic plate behind rigid square body	Wall^16^; Wall and Ramm^17^; Hübner et al^18^	204, 333		Transient
2D	Modified lid‐driven cavity	Wall^16^; Mok^19^	200		Transient
2D	Elastic plate behind rigid cylinder	Turek and Hron^20^	20, 100, 200		Both
2D	Elastic plate with rear mass behind rotary cylinder	Gomes and Lienhart^21^, ^22^, ^25^; Gomes et al^23^; Gomes^24^	140, 195, 15400	X	Transient
2D	Elastic plate with rear mass behind rigid cylinder	Kalmbach and Breuer^26^; Kalmbach^27^	30470	X	Transient
2D	Elastic plate behind rigid cylinder	Kalmbach^27^; De Nayer et al^28^	30470	X	Transient
3D	Shell in steady‐state cross‐flow	Bathe and Ledezma^29^	0 − 5000		Steady
3D	Elastic plate behind truncated cone	Kalmbach^27^; Kalmbach et al^30^	21300 − 32000	X	Transient
3D	Elastic structure in merging flow from two inlets	*this work*	651, 1283	X	Both

Abbreviation: FSI, fluid‐structure interaction.

We emphasize that the list is not exhaustive, see, for example, previous works.^28^, ^29^

While verification and validation testing remains critical, extension to biomedically relevant problems in three dimension remains limited. To date, most benchmarks favor using water or highly viscous fluids. This leads to either high (15 400‐32 000) or low (20‐333) Reynolds (*R* *e*) number flows. These conditions are in contrast to those observed in many biomedical FSI applications, with flows ranging from 100 to 3000 for a typical medium‐/large‐sized artery.^31^ Only a few experimental and numerical works consider FSI in three dimensions, with most test cases derived for fast numerical testing in two dimensions. However, fluid flow and solid deformation in biomedical problems generally require 3D analysis, making the augmentation of current 2D benchmark tests with 3D tests an appealing choice for further verification and validation. Further, as data derived from clinical imaging techniques are frequently used in FSI modeling frameworks, a test case that uses medical imaging for data acquisition provides an appealing choice for examining simulation accuracy under typical conditions.

**Figure 1:**
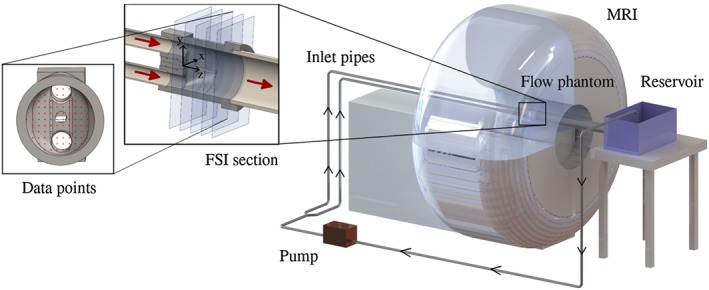
Schematic drawing of the experimental setup: a centrifugal pump (EWP80, Davies Craig, Victoria, Australia) pumps fluid through a flexible tube (length 1m, inner diameter (ID) ⊘19mm) via a Y‐junction and two flexible tubes (6m, ID ⊘19mm) connected to rigid straight inlet pipes (2m, ID ⊘21.9mm) running along the MRI bed into the flow phantom. The closed loop is completed with a larger outlet pipe (1.5m, ID ⊘76.2mm), a reservoir and flexible tubing (11m, inner diameter ⊘19mm) connecting the reservoir and the pump. The FSI section consists of two inlets, a silicone filament attached to the wall and a single outlet. Further, equispaced data points for comparison of experimental data and numerical results on multiple planes are shown

In this work, a 3D biomedically motivated FSI experiment is presented, with the aim of providing a new reference for testing of numerical FSI models. This was achieved by selecting flow rates yielding moderate *R* *e* numbers, use of viscous fluids closer to that of blood, and use of nonlinear soft solid materials with suitable material properties. Imaging data for driving forward models was acquired using phase contrast magnetic resonance imaging (PC MRI), as has become common in patient‐specific biomedical applications. A precise definition of the geometry was obtained through the use of computer‐aided design (CAD) generated 3D printed geometry. Straightforward boundary conditions can be derived from measurement of inlet conditions along with devising the experiment to have simple‐to‐define outflow and fluid‐solid conditions. Laminar flow was measured for both steady‐state and transient test cases with *R* *e* numbers of 651 and 1283, respectively (on the basis of maximum inflow velocity and inlet diameter); reflecting *R* *e* numbers found in the cardiovascular system (for example, *R* *e* numbers of order of 100 to 1000 for medium‐sized artery^31^). Detailed measurements of the flow were quantified using PC MRI throughout the flow domain providing steady and transient measures of both fluid/solid motion. Mesh data along with quantitative information for verification and validation is provided and forms the basis of a 3D FSI Benchmark Challenge *
http://cheart.co.uk/other-projects/fsi-benchmark/

32 aimed at assessing various FSI models and algorithms.

In Section 2, the experimental design is presented focusing on the portion of the experiment where FSI occurs. Material selection and properties are detailed and motivated, data acquisition protocols are defined, and experimental measurement errors quantified. In Section 3, experimental data are presented for both steady and transient test cases. Fluid/solid motion is quantified, and important flow and deflection patterns are highlighted. The experimental design and acquired data are discussed in Section 4.

## 2 EXPERIMENTAL DESIGN AND DATA ACQUISITION

CAD tools were employed for precise geometry definition using SolidWorks.^33^ An MRI compatible experimental flow phantom model was developed and embedded into an experimental test rig including pump and fluid reservoir (see Figure 1). The relevant section for recording FSI in the flow phantom (referred to as FSI section) was positioned at the isocenter of the bore of an MRI scanner. Two test cases were established with constant (phase I) and oscillating (phase II) inflow, yielding steady and oscillatory motion of an elastic structure within surrounding flow, respectively.

The following sections focus on the design of the FSI section including geometry and material selection of the fluid and solid domains. Further, data acquisition is detailed, including measurement of material properties, image‐based flow/geometry data and experimental errors.

### 2.1 FSI section

Numerical experiments were performed in 2D and 3D to review and optimize geometry and experimental design and explore suitable sets of materials. The final design of the FSI section comprises of flow entering the flow phantom via two inlets, merging and leaving through the outlet pipe (see Figure 1). A solid is mounted in the merging region that extends downstream into the flow domain. The model is oriented, in such a way that the z‐axis is aligned with the main flow direction and the y‐axis opposes the direction of gravitational forces. The origin of the considered right‐handed coordinate system is at the center of the attachment point of the elastic solid at the fluid domain wall.

Design of the model aimed to avoid sharp edges, kinks or gaps and further allow straightforward implementation of boundary conditions in the numerical setting (for example, *no‐slip*/*no‐displacement* at fluid/solid domain wall). A CAD‐defined geometry^33^ of the fluid domain was converted into a flow phantom model (see Figure 1), which was then 3D printed (ProJet HD 3000Plus, 3D Systems, California, USA; acrylic plastic, VisiJet® EX200, 3D Systems, California,USA) at a resolution of 750 × 750 × 890 DPI(thus, thickness of printed layers being 29 *μ*m). The surface was smoothed and polished to remove staircase effects. Inlet and outlet pipes were selected with compatible inner diameters to avoid disturbance of flow. The distance between inlets and outlet of the flow phantom was 119 mm. A cylindrical insert held a solid body and slid into a prepared hole in the flow phantom, such that it was level with the flow phantom wall. The insert was designed using CAD^33^ and 3D printing.

**Table 2:** Selection of solid material models and parameters obtained from nonlinear least‐square fit

**Material model**	**Predicted force**, N	**Parameters**, Pa
Linear‐elastic	*c*_1_ *A*_0_(*λ* − 1)	*c*_1_ = 221598.05
Blatz‐Ko^42^, ^43^	*c*_1_ *A*_0_(*λ* − *λ* ^ − 2*ν* − 1^)	*ν* = 0.463
		*c*_1_ = 98414.81
Neo‐Hookean^44^	*c*_1_ *A*_0_(*λ* − *λ* ^ − 2^)	*c*_1_ = 96865.09
Mooney‐Rivlin^44^	*c*_1_ *A*_0_(*λ* − *λ* ^ − 2^) + *c*_2_ *A*_0_(1 − *λ* ^ − 3^)	*c*_1_ = 103533.82
		*c*_2_ =− 8891.65
Ogden^44^	$A_{0}\;\sum_{i=1}^{3}c_{i}(\lambda^{d_{i}-1}-\lambda^{-d_{i}/2-1})$	*c*_1_ = 999994.27
		*c*_2_ = *c*_3_ = *c*_1_
		*d*_1_ = *d*_2_ =− 0.10
		*d*_3_ = 0.76

Here, *A*_0_ is the reference area, and *λ* is the experimental stretch. Note that if the parameter optimization is constrained by *c*_*i*_ ⩾ 0, the Mooney‐Rivlin model reduces to the Neo‐Hookean model (eg, *c*_2_ = 0).

**Table 3:** Experimentally determined fluid and solid material parameters

		**Gravitational**	**Fluid**	**Dynamic**	**Kinematic**	**Solid**
	**Temperature, °C**	**acceleration, m/s** ^2^	**density, kg/m** ^3^	**viscosity, mPa·** **s**	**viscosity, mm^2^/** **s**	**density, kg/m** ^3^
Phase I	23.6	9.81	1163.3	12.50	10.75	1058.3
Phase II	22.0	9.81	1164.0	13.37	11.48	1058.3

For simplicity, a brick‐shape geometry was selected for the solid. Its relevant volume protruding into the fluid domain was 11 × 2 × 65 mm^3^, with its long axis being aligned with the z‐axis and its short axis being aligned with the y‐axis.

### 2.2 Material selection and material properties

A key aspect of the experimental design was the selection of the fluid. To minimize the potential for turbulence, increase shear stresses, and maintain test cases with steady and transient FSI, an aqueous glycerol solution^34^, ^35^, ^36^, ^37^ was selected with a mixing ratio of approximately 65%wt glycerol/35%wt water. Depending on mixing ratio and temperature, the viscosity of the Newtonian fluid can range from approximately 1 to 1400 times the viscosity of water. Therefore, fluid shear stresses exerted onto the solid can be increased while reducing the *R* *e* number. The temperature of the aqueous glycerol solution within the fluid reservoir was recorded during the FSI experiment using a calibrated electronic thermistor thermometer. This allowed us to ensure that rheological measurements of the solution were calibrated with the temperature of the experiment. We note that the fluid temperature varied between phases I and II of the experiment (as these were conducted on different days); however, recorded fluctuations for each of the test cases were negligible. Fluid density and viscosity were determined (see Table 3) using a Stabinger viscometer (SVM 3000, Fa. Anton Paar, Graz, Austria) for each experimental temperature.

**Figure 2:**
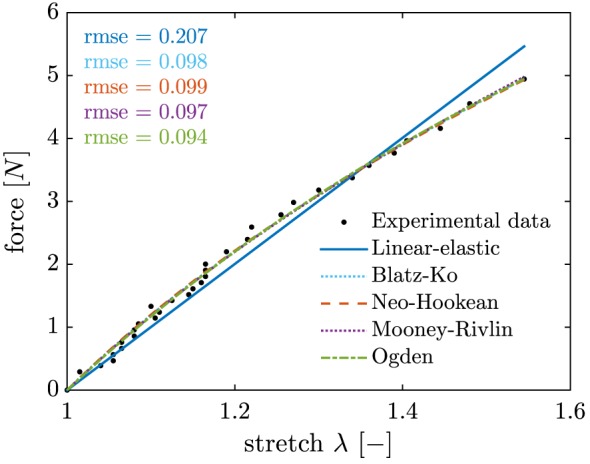
Uniaxial tensile load‐displacement test data and fitted material models, see Table 2

Further, selection of a silicone material (RTV Silicone Rubber 13 Shore A, Tomps, UK) aimed at minimizing stiffness of the material. Thus, forces required to drive solid motion and deformation are minimized. Density of the silicone material was measured using Archimedes' method^38^ and found to be about 9*%* smaller than the density of the fluid, see Table 3. To characterize mechanical behavior of the silicone material and to assist in the selection of an appropriate constitutive model, uniaxial tensile load‐displacement test data^39^ were obtained (for loading corresponding to elongation of the sample from 20.0 to 30.9 mm) and are provided in Figure 2.

The silicone material used can be approximated as incompressible^40^ and it is known from Bondi,^41^ that it can be modeled using a one‐parameter Blatz‐Ko material model.^42^, ^43^ However, additional models from Bower^44^ were considered (assuming perfect incompressibility of the material) and respective material parameters obtained using a nonlinear least‐square fit of the analytical form to the uniaxial tensile load‐displacement test data (see Figure 2 and Table 2).

Note, experiments for phases I and II were performed at different time instances. Because the used silicone material undergoes a continuous curing process, stiffness properties may change. Therefore, additional measurements were performed to enable calibration of material models employed, see Section 2.3.

The flow evolving over one cycle was recorded at ten slices along the z‐axis of the flow phantom (spacing 10 mm) for 25 time frames, eg,

(1) $$t_{n}=\left\{\begin{array}{ll} 0.073+0.216\cdot (n-1), & n=1,\text{⋯},15,\\ 3.917+0.216\cdot (n-16), & n=16,\text{⋯},25. \end{array}\right.$$

We note, that the number of signal averages per recording was six, that is, each acquired image is an average of six images. Further, individual VENCs were selected for each velocity component. Each component scan took 22 minutes.

Under zero flow conditions, the silicone filament exhibits a deflection in positive y‐direction because of a difference in fluid/solid density. Because phases I and II of the FSI experiment were performed at different points in time and considering the aforementioned potential change in silicone stiffness, deflection of the silicone filament was recorded under zero flow conditions to enable calibration of solid material models. This was done as part of the acquisition of geometry data in an image stack to obtain the orientation of the model.

**Table 4:** The MRI sequences employed for acquisition of geometry and flow data. Details on spatial and temporal resolution of T1 turbo field echo (TFE) and turbo spin echo (TSE) sequences, image stack size and selected VENC values

**Phase**	**Sequence**	**Scan**	**Image plane**	**Image size**	**Voxel size**, mm^3^	**Number of time frames**	**Scan duration**, s	**VENC, cm/s**
I	T1 TFE	geometry (no flow)	*y* *z*	40 × 256 × 256	2 × 0.977 × 0.977	1	138	—
	T1 TFE	geometry (flow)	*y* *z*	40 × 256 × 256	2 × 0.977 × 0.977	1	138	—
	T1 TFE	inlet, flow field, *v*_*x*_	*x* *y*	192 × 192 × 35	1.302 × 1.302 × 6	1	151	12
	T1 TFE	inlet, flow field, *v*_*y*_	*x* *y*	192 × 192 × 35	1.302 × 1.302 × 6	1	155	12
	T1 TFE	inlet, flow field, *v*_*z*_	*x* *y*	192 × 192 × 35	1.302 × 1.302 × 6	1	114	80
II	TSE	geometry (no flow)	*y* *z*	25 × 512 × 512	4 × 0.781 × 0.781	1	348	—
	T1 TFE	inlet, *v*_*x*_	*x* *y*	192 × 192 × 1	1.302 × 1.302 × 10	28	132	5
	T1 TFE	inlet, *v*_*y*_	*x* *y*	192 × 192 × 1	1.302 × 1.302 × 10	28	132	5
	T1 TFE	inlet, *v*_*z*_	*x* *y*	192 × 192 × 1	1.302 × 1.302 × 10	44	132	40
	T1 TFE	flow field, *v*_*x*_	*x* *y*	192 × 192 × 10	1.302 × 1.302 × 10	25	1320	12
	T1 TFE	flow field, *v*_*y*_	*x* *y*	192 × 192 × 10	1.302 × 1.302 × 10	25	1320	12
	T1 TFE	flow field, *v*_*z*_	*x* *y*	192 × 192 × 10	1.302 × 1.302 × 10	25	1320	40

During phase I of the experiment, the pump was set to a constant pump rate of approximately 14.07 l/min. Selection of the pump rate aimed at increasing the observed deflection from the zero flow state while maintaining a low Reynolds number. Preliminary flow experiments and numerical investigations showed no solid motion, such that data could be acquired assuming steady‐state flow. In fact, violation of the steady‐state assumption would cause image blur in the magnitude images because of time averaging of fluctuating velocities causing artifacts (in particular near the solid). This, however, was not observed. Further high frequency (approximately 30 Hz) low‐quality datasets were acquired confirming no solid motion and supporting validity of the steady‐state assumption.

Firstly, the position of the deformed silicone filament was recorded under flow conditions using a T1 TFE flow sequence. Secondly, flow images were acquired using individual image stacks for each velocity component. Here, care was taken to select an appropriate velocity encoding sensitivity (VENC) value for each component scan. This is to avoid velocity aliasing when real flow velocities exceed the VENC value and an incorrect phase is assigned. On the other hand, setting a much larger VENC was avoided, because the work of Lotz et al^47^ reports that errors tend to be ⩽10*%* of the selected VENC if the selected VENC value is three times as large as the expected velocity. Thus, the VENC value was tuned for each velocity component for improved image accuracy, see Table 4.

During phase II of the experiment, the pump was set to an oscillating pump rate with approximate maximum pump rate of 7.71 l/min. The period of the experiment was controlled by the scanner, with flow sequences modified to trigger low frequency current pulses from the scanner to the pump using a custom‐built current amplifier. Thus, any delays in the image acquisition process could not accumulate and lead to out‐of‐sync data acquisition. Pulsatile inflow and a repeatable deflection pattern of the silicone filament were observed in preliminary survey scans, such that a periodic steady‐state was assumed for final data acquisition. Similarly to phase I, violation of this assumption would result in blurring in the magnitude image, which was not observed. Again, further high frequency (approximately 30 Hz) low‐quality data were acquired confirming periodic solid motion.

Velocity components were recorded individually at a single plane crossing both inlets of the flow phantom to provide boundary condition data for the numerical setting. Inflow scans were acquired at 28 time frames for *v*_*x*_ and *v*_*y*_ and 44 time frames for *v*_*z*_ using phase‐contrast MRI sequences. The reduced VENC for non‐axial velocity components yielded a longer acquisition time per time frame^48^ thus the reduced number of scans.

### 2.3 Image‐based data acquisition

MRI techniques were employed to acquire both geometry and flow data. A clinical 3T MRI scanner was used for data collection (Philips Achieva, Philips Healthcare, Best, the Netherlands). For imaging of flow, 3T MRI scanners are generally superior to 1.5T MRI scanners because of a higher signal‐to‐noise ratio (SNR).^45^, ^46^ The MRI sequences were selected to acquire geometry data under zero flow and flow conditions, inflow boundary condition data and flow images in image stacks. Additionally, temporal resolution was added for phase II to acquire data throughout a full cycle. Details on employed sequences can be found in Table 4.

Once acquired, scans were first registered to determine the mapping between the MRI coordinate frame and the model coordinate frame (see Figure 1). Motion of the MRI table was restricted during acquisition, enabling use of all images to be used when determining the relation between MRI and model coordinates during each phase. The origin was found by examining the geometry scans, and any rotation about the y‐axis was eliminated by ensuring cross‐sectional conformity in the magnitude images (flow stacks). Once registered, MRI data at the inlets were processed by fitting parabolic profiles to both upper and lower inlets (for all time frames) to simplify representation of inflow conditions (for a multifrequency Womersley solution fit for phase II, see Appendix A). At all other slices, flow was extracted and coordinate positions recorded.

**Figure 3:**
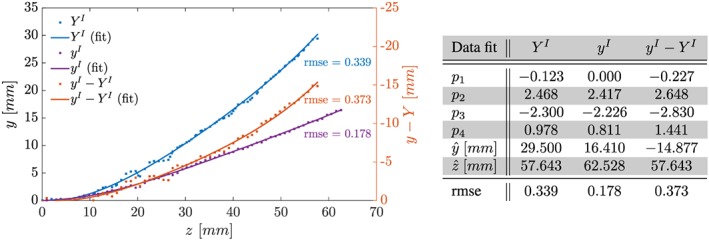
Phase I, recorded position of centerline of solid at x = 0 mm under zero inflow conditions, Y ^I^, subject to constant inflow, y ^I^, and relative displacement, y ^I^ − Y ^I^. Shown are data points extracted from MRI data and polynomial fits of order 4, $y(z)=ŷ\;[p_{1}(z/\hat{z})+p_{2}{\left(z/\hat{z}\right)}^{2}+p_{3}{\left(z/\hat{z}\right)}^{3}+p_{4}$ ${\left(z/\hat{z}\right)}^{4}]$, $z\in [0,\hat{z}]$. Coefficients and root‐mean‐square error (rmse) as given in Table

**Figure 4:**
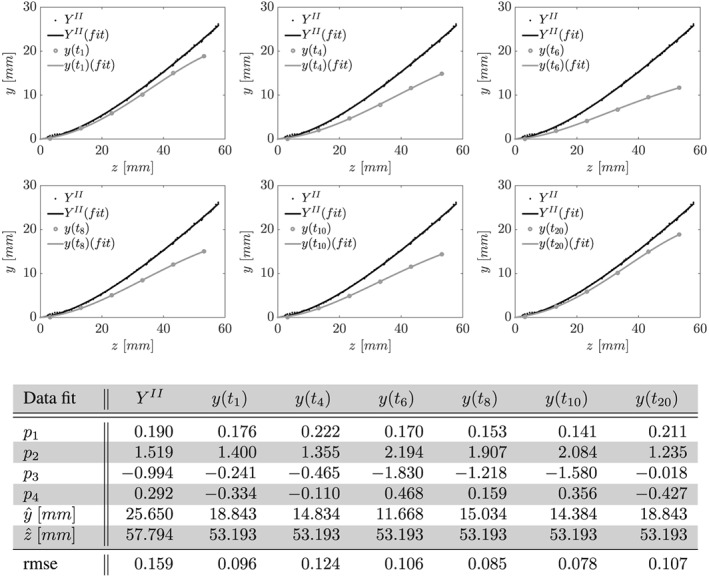
Phase II, recorded position of centerline of solid at x = 0 mm under zero inflow conditions, Y ^II^, and subject to period inflow, y(t), at t ∈ {t_1_,t_4_,t_6_,t_8_,t_10_,t_20_} (see Equation 1). Shown are data points extracted from MRI data and fit polynomial of order 4, $y(z)=ŷ\;[p_{1}(z/\hat{z})+p_{2}{\left(z/\hat{z}\right)}^{2}+p_{3}{\left(z/\hat{z}\right)}^{3}+p_{4}{\left(z/\hat{z}\right)}^{4}]$, $z\in [0,\hat{z}]$. Coefficients and root‐mean‐square error (rmse) as given in Table

The position of the silicone filament for steady‐state cases was extracted from multiple isochronous anatomical image slices (yz‐planes). Five and three image slices were processed for phases I and II, respectively. Image slices were cropped to focus on the region containing the silicone filament and the origin identified. For each slice, intensity minima were detected row‐ and column‐wise and averaged. Finally, these results were averaged and a polynomial of order 4 fit to the data (see Figures 3 and 4).

Silicone filament motion and deformation was observable within the magnitude of flow images for periodic steady‐state cases. The position of axial points for each time frame were determined from image slices in the xy‐plane by detecting intensity minima for each velocity component image and averaging the individual results. Again, a polynomial of order 4 was fit to the data (see Figure 4).

We note that the resulting motion data are not Lagrangian but represent the intersection of the centerline of the silicone filament with xy‐planes at fixed positions.

During preliminary experiments, pressure measurements inside the FSI section were obtained using a catheter. Unfortunately, disturbance of the flow field and artifacts in the acquired MRI data could not be fully avoided. Thus, pressure recordings were omitted during the two phases of data acquisition. Further, Hessenthaler et al^49^ showed that the hydrostatic pressure component can be removed from the modeled FSI system via a substitution in the pressure variable, such that pressure measurements are not imperative.

#### 2.3.1 Experimental measurement errors

Measurement error in the velocity data extracted from PC MRI can be challenging and is often not determinable from the images directly. While noise in the velocity measurement (*σ*) can be related to the SNR and VENC, $\sigma =\sqrt{2}\;\text{VENC}/(\pi \;\text{SNR})$, a myriad of additional artifacts can also impact measurement accuracy.^50^ However, Khodarami et al^51^ using the same scanner and sequences used in the current work reported errors of less than 5*%* when comparing velocity measures between PC MRI and stereoscopic particle image velocimetry (PIV).

Further, precise geometry definition was achieved using CAD tools, and experimental design aimed to minimize errors. For example, fluid temperature was recorded during the experiments to improve measurement accuracy of fluid material parameters. Nevertheless, measurement errors occurred, and manufacturing tolerances existed. For example, the uniaxial tensile load‐displacement test to determine mechanical response was conducted manually by measuring length change with incremental loading. Known weights were added to the sample to cause elongation. Thus, measurements accuracy depended on the accuracy of the used Vernier caliper and balance, and the relative error is larger at small stretches. A list of known sources of experimental error is given in Table 5 along with error intervals.

**Table 5:** List of measurement error intervals for different tools and devices used

	**Method**	**Impact**	**Experimental error**
SolidWorks meshing	SolidWorks in‐built mesh generator parameter: meshing resolution tolerance	Geometry	± 0.011 mm
3D printing accuracy	Provided by manufacturer	Geometry	± 0.025 − 0.05 mm
3D model smoothening	Sandpaper 500, 1000 grit size parameter: estimated max error due to material removal	Geometry	− 0.1 mm
Silicone filament dimensions	Vernier calipers	Geometry	± 0.2 mm
Silicone density	Archimedes suspension method,^38^ error estimated as 95% confidence interval of 12 measurements	Solid density	± 0.014 g/cm^3^
Fluid density	Stabinger viscometer	Fluid density	± 0.0005 g/cm^3^
Fluid dynamic viscosity	Stabinger viscometer	Fluid dynamic viscosity	± 0.35*%*
Fluid kinematic viscosity	Stabinger viscometer	Fluid kinematic viscosity	± 0.35*%*
Tensile testing ‐ displacement	Vernier calipers	Solid mechanical response	± 0.2 mm
Tensile testing ‐ load	Incremental water volume injected by 20 ml syringe	Solid mechanical response	± 0.1 g
Flow velocity	MRI measurements (see Table 4)	Velocity magnitude	< 0.05· *VENC*
Solid position	MRI measurements (see Table 4)	Geometry	Voxel size

## 3 RESULTS

To illustrate the FSI phenomena observed in phases I and II of the experiment, a qualitative description of steady and transient solid motion and fluid flow is given in this section. Experimental results extracted from imaging data are presented. We note that extracted experimental values are available in the online supplement.

### 3.1 Phase I

Under zero inflow conditions, the silicone filament exhibited a steady deflection (approximately 29.50 mm, see Figure 3) because of buoyancy forces. Increasing the inflow velocity decreased the peak deflection for moderate inflow velocities because of surrounding flow and fluid shear stresses being exerted onto the solid. Further, increases in inflow led to little change in deflection until the flow became turbulent, at which point the filament exhibited unsteady deflection. Data were recorded with peak inflow velocity values of 630 and 615 mm/s for the upper and lower inlet, respectively. Observed profiles were parabolic in nature as expected on the basis of Poiseuille flow in a cylindrical tube. The steady silicone filament deflection under constant inflow conditions was approximately 16.41 mm in the *y*‐direction, see Figure 3, whereas no significant deflection was observed in the *x*‐direction.

**Figure 5:**
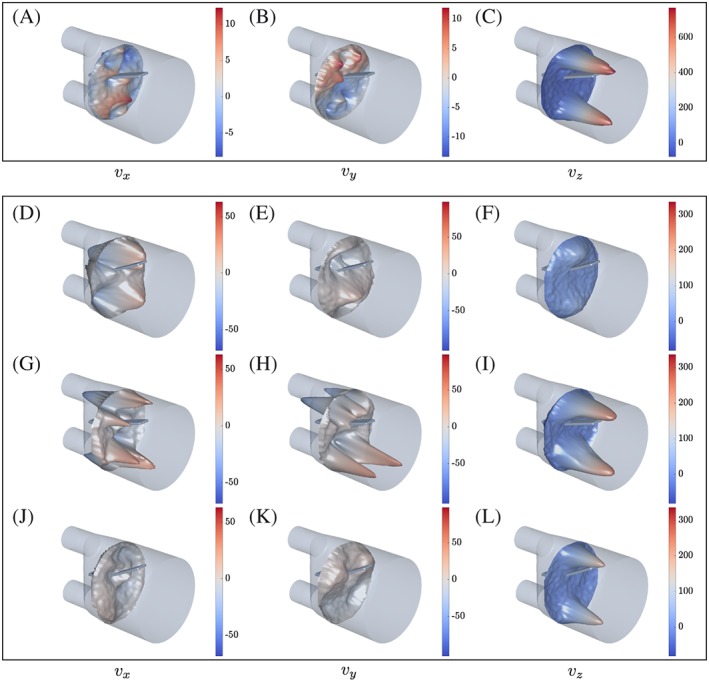
Recorded flow velocity components for A to C phase I at z ≈ 30 mm, and D to L phase II at z ≈ 33 mm, t ∈ {0.721, 1.153, 2.665} s. Velocity components v_x_ are given in the left column, v_y_ in the middle column, and v_z_ in the right column. Note that data points **d** ^i^ are shown at displaced coordinates **d** ^i^ + [v_x_, v_y_, m·v_z_]^T^·1s with m = 0.07 and m = 0.2 for phases I and II, respectively

Flow in the FSI section was principally oriented down the z‐axis of the model because of the jets entering from the inlets. We note the two distinct peaks in the *v*_*z*_ component on given planes parallel to the xy‐plane, see Figure 5. Further, symmetries are observed in the *v*_*y*_ and *v*_*z*_ components with respect to the yz‐plane (see Figure 5).

### 3.2 Phase II

**Figure 6:**
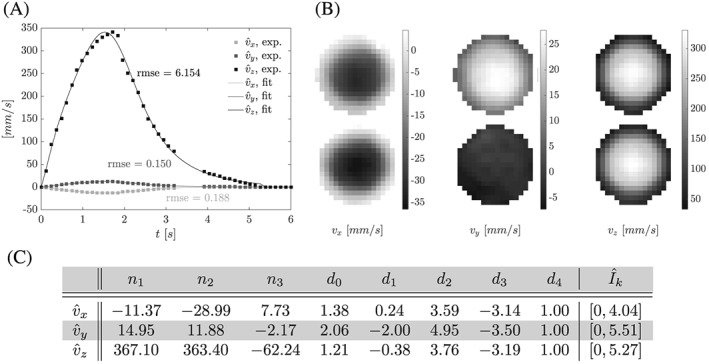
Phase II, recorded inflow boundary condition data. A, Recorded peak inflow and fit curves, see C. B, Raw image data (cropped at flow phantom wall) of inlet velocity profiles at peak inflow. C, Coefficients for ${\hat{v}}_{k}(t)=[\sum_{i=1}^{3}n_{i}t^{i}]/[\sum_{j=0}^{4}d_{j}t^{j}]$ for $t\in {Î}_{k}$, k ∈ {x, y, z}, fit to recorded peak inflow with ${\hat{v}}_{k}(t)=0$ for $t\in I\backslash {Î}_{k}$. Note that flow in the v_y_ direction was observed only in the upper inlet

Under zero inflow conditions, the silicone filament exhibited a steady deflection as shown in Figure 4. At the tip of the silicone filament, the deflection is approximately 13*%* smaller than recorded under zero flow conditions for phase I of the FSI experiment. Periodic flow generated by the pump resulted in pulsatile inflow with frequency *f* = 1/6 *H* *z*(see Figure 6). Peak values for each time frame were obtained on the basis of a best fit to a parabolic profile. A continuous representation of the fitted peak values per component is given in Figure 6. In the following, we refer to a given cycle with time *t* ∈ *I*: = [0, *T*] and a duration of *T* = 6 s.

**Figure 7:**
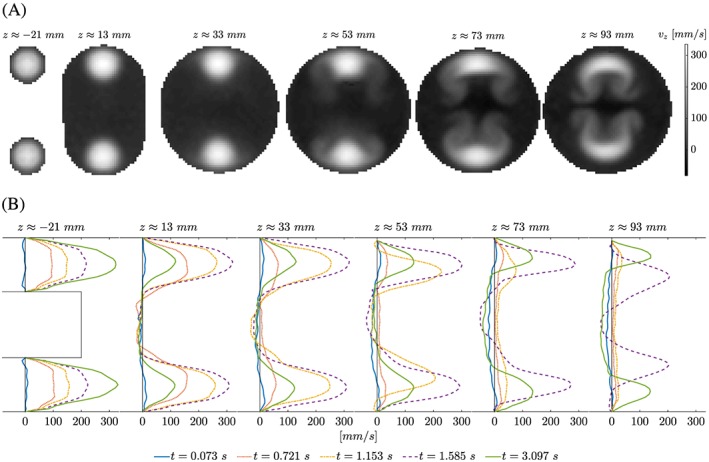
Phase II, flow velocity component v_z_ at z ≈ { − 21,13,33,53,73,93} mm. A, Raw image data (cropped at flow phantom wall) of flow jets entering the FSI section at the two inlets create a double Ω‐shaped flow pattern downstream at time t ≈ 2.017 s. B, At x = 0 mm (midplane), velocity profiles are shown for multiple time frames. It is noted that peak values of flow are observed at different time instants and that reflow regions develop near the long axis of the flow phantom model

During the first half of each cycle, flow in the FSI section was principally oriented down the z‐axis of the model because of flow jets entering from the two inlets at high velocity. Minor flows were measured in the other velocity components, see Figure 5. Once flow decelerated in the second half of each cycle, the ratios $\max\left|v_{z}\right|/\max\left|v_{x}\right|$ and $\max\left|v_{z}\right|/\max\left|v_{y}\right|$ become smaller. Figure 7A shows how flow patterns changed between image planes for one time frame. For example, two distinct peaks in *v*_*z*_ were present at z ≈ 33 mm. On the other hand, a double *Ω*‐shaped flow pattern was observed for *v*_*z*_ at z ≈ 93 mm. Figure 7B illustrates how flow develops at *x* = 0 mm and multiple slices along the z‐axis for multiple time frames. Note, at *x* = 0 mm peak values in *v*_*z*_ were observed at different time frames for different *z*‐positions. Further, back‐flow occured at multiple *z*‐positions and time frames pushing the silicone filament upwards.

The silicone filament follows a repeatable deflection pattern, see Figure 4. From its resting position at the beginning of each flow cycle, it is deflected in negative *y*‐direction by the incoming flow wave. Once flow decelerates, gravitational forces become more dominant and the silicone filament moves back upwards. It performs a swing before reaching its initial position. Note that the resting position under zero flow conditions is significantly different to the position of the silicone filament at the beginning of each flow cycle. Similar to phase I, no significant deflection in the *x*‐direction was observed.

## 4 DISCUSSION

The FSI experiment presented in this paper provides new means of testing FSI algorithms. It extends previously proposed numerical benchmark problems and FSI experiments to a 3D setting with flow in the laminar regime.

Key characteristics of the FSI experiment were designed to be comparable with those of many biomedical engineering problems. For example, flow rates were selected such that resulting flow was in the laminar regime. The chosen fluid is moderately viscous and incompressible, and the solid material exhibits nonlinear incompressible material characteristics. Importantly, MRI has been used for data acquisition as it is often used in translational biomedical engineering applications.

### 4.1 Overview

Overall, a comprehensive set of MRI data was acquired quantifying flow and solid motion at multiple slices through the FSI section with temporal resolution (for phase II, see Equation 1). Preliminary experiments indicated steady and periodic behavior of the FSI system, such that assumptions of a steady‐state and a periodic steady‐state are justified during data acquisition in phases I and II of the FSI benchmark experiment. Phase I of the FSI experiment yielded a stable deflection of the solid within a steady‐state flow field, such that dynamic components become negligible for FSI models. Here, modeling of the steady‐state scenario is tested with a focus on the correct prediction of the pressure distribution because shear stresses are less important than for dynamic FSI problems. Testing also includes the correct spatial prediction of solid position and flow as well as testing of the coupling scheme.

On the other hand, phase II gave periodic solid motion within a periodic steady‐state flow field. As opposed to phase I, modeling of the dynamics of the fluid and the solid as well as the coupled FSI problem becomes of prime importance. Significant variation in solid motion is observed as well as changes to flow dynamics. This phase thus presents additional challenges (for example, coupling of time integration schemes for both subsystems) on top of the first phase.

### 4.2 Online supplement

Postprocessed data were presented in this paper and are available in the online supplement. This supplement contains surface geometries (CAD), tensile load‐displacement test data, and velocity boundary condition data in tabulated format. Further, data files containing extracted fluid/solid velocity and solid position for a set of equispaced data points on multiple slices through the FSI section.

### 4.3 3D FSI Benchmark Challenge

On the basis of the experiment presented in this work, a 3D FSI Benchmark Challenge was initiated.^32^ Geometry and boundary condition data, as well as material parameters and measurement data characterizing solid mechanical behavior were supplied to interested FSI research groups. Participants were asked to model the FSI problem and predict fluid/solid velocity and solid displacement using a simulation environment of their own choice to ultimately compare various model assumptions and FSI algorithms for prediction capability, accuracy, computational cost, and others. For further details, see the work of Gaddum et al.^32^

### 4.4 Limitations

The experimental design was optimized, and additional data acquired to minimize measurement errors and assist with correct modeling of the FSI problem. Of course, measurement errors could not be fully avoided. For example, tolerances during manufacture (eg, 3D printing) and measurement (eg, viscometer) impact precision as outlined in Table 5.

State‐of‐the‐art modeling frameworks that are used for patient‐specific models increasingly integrate data obtained from clinical imaging techniques,^9^, ^52^, ^53^, ^54^ such as MRI. In general, MRI measurements of flow velocity and solid position represent volume and time averages (due to finite voxel size and acquisition time). In this sense, steady‐state and periodic steady‐state assumptions were made and found valid, as no blurring was found in acquired image data. Violation of slice‐to‐slice mass conservation was found, likely because of measurement errors and distance from the bore isocenter. For example, measurement errors in recorded flow fields are generally a fraction of the selected VENC value, such that the relative error increases with decreasing flow velocity in unsteady flow cases. Nevertheless, due to optimizing VENC values for each flow velocity components, measurement errors could be minimized. It is noted, that flow velocities and respective volume averages were found to be sufficiently similar in numerical experiments, such that comparability of volume averaged experimental data and point‐wise numerical results is expected. The representation of inflow boundary conditions by parabolic profiles for each time frame is an approximation to simplify prescription of boundary conditions in the numerical setting. A modest improvement of the description of the inflow can be achieved by fitting the multifrequency Womersley solution (see Appendix A). Here, we minimized the *L*_2_‐norm of the radial profile with the multifrequency Womersley solution to determine the unknown driving coefficients.

The use of MRI also constrained the experimental design. For example, all parts exposed to the magnetic field needed to be MRI compatible, and the requirement for a periodic steady‐state was essential for data acquisition. The temporal resolution of MRI sequences used was also limited to avoid prolonged data acquisition. In this regard, PIV may provide better performance and would be less prone to averaging effects than MRI because of the underlying technology of tracing particles to obtain an instantaneous velocity map. Nevertheless, in our case temporal resolution of the employed MRI scanner was sufficient, and the employed sequences allowed us to record all three spatial velocity components whereas standard 2D PIV is limited to imaging in‐plane velocity components. An additional advantage of MRI over PIV is the lack for optical requirements, for example, accounting for the refraction index of the flow phantom material, as well as the less intrusive approach (no tracer particles required). Further, MRI tends to provide the input data commonly used in biomedical engineering research geared toward translation. Hence, the definition of input data and comparison of end results are considered a valuable step for translational FSI applications. For further details on PIV, see previous works.^55^, ^56^, ^57^ For a comparison of PC MRI and PIV for the investigation of an intracranial aneurysm phantom, see previous works.^58^, ^59^

Gravitational forces make an important contribution to the mechanism that is employed to obtain a repeatable deflection pattern (see Section 3.2) as it is the main driving force of the solid motion observed during the second half of each cycle. To yield such behavior, however, the frequency of the oscillating flow rate was selected to be relatively small as compared to the normal heart rate of an adult at rest, which ranges from 1 to 1.7 Hz. Therefore, future improvements or test cases could be obtained by investigating frequencies more closely related to human heart rate, eg, by employing a different FSI mechanism.

In the current study, a uniaxial tensile load‐displacement test was conducted manually. Although a device‐based test would be preferable, the data derived represent an adequate sampling to observe the material behavior. Moreover, due to the continuous curing process, calibration to the zero flow deflection data is recommended for estimating solid model material parameters in phases I and II. We note that the solid material exhibits viscoelastic behavior. However, viscous effects are minimal at low frequency and were thus deemed negligible in this study.

## 5 CONCLUSION

In this paper, an FSI experiment for validation of FSI modeling frameworks was presented, complementing and extending well‐known standard numerical and experimental FSI tests. Focus of the experiment was on aspects encountered in typical biomedical engineering applications. For example, flow was in the laminar regime with biologically relevant Reynolds numbers. A moderately viscous incompressible Newtonian fluid interacts with a nonlinear incompressible isotropic solid in a fully 3D setting under the influence of gravitational forces. Like in many biomedical engineering applications, MRI was used for acquisition. A comprehensive data set comprising of geometry and flow images quantifying fluid and solid motion in space and time was obtained and is made available in the online supplement.

## DATA ACCESSIBILITY

## Supporting information

supporting info item

## APPENDIX A

### A.1

Once the inflow boundary scans were acquired and registered, the centers of either inlet were identified. Then, a multifrequency Womersley solution was fit in a local coordinate frame (with respect to radius *r*, ie, distance to center point) for each velocity component individually,

(A1) vi(r,t)=a0·1−r2R2+∑m=1M−1Re{am·1−J0(λm·r)J0(λm·R)exp(ıwmt)},

for (*r*, *t*) ∈ [0, *R*] × [0, *T*] and *i* ∈ {*x*,*y*,*z*}, with complex fitting parameter *a*_m_, radius *r*, pipe radius *R*, $\text{ı}=\sqrt{-1}$, *w*_m_ = m*π*/*T*, $\lambda_{m}={\text{ı}}^{\;3/2}\sqrt{w_{m}/\nu }$, number of frequencies *M* and *J*_0_(·) denoting the Bessel function of order zero.

The fit was obtained as follows. Firstly, we interpolated the data at 100 equispaced time points. Secondly, the mean square error at each data point was weighted by the variance of the data as well as the number of data points per radius *r*. Then, we employed a quasi‐Newton algorithm to minimize the weighted mean square error.

For *M* = 10, we obtained the fitting parameters given in Table 6. In this case, the root‐mean‐square errorsof the individual fits are within 5% of the respective root‐mean‐square errors of fits with *M* = 50. Further, the weighted mean square error of the parabolic fits (see Section 2.3) was up to 4% larger compared to the fit on the basis of the Womersley solution for *M* = 10.

**Table 6:** Complex parameters a_m_ in multifrequency Womersley solution (see Equation A1), defining inflow profiles v_x_(r, t), v_y_(r, t) and v_z_(r, t) (left to right) for y > 0 (top row) and y < 0 (bottom row)

	*m*	Re{*a*_*m*_}	Im{*a*_*m*_}	*m*	Re{*a*_*m*_}	Im{*a*_*m*_}	*m*	Re{*a*_*m*_}	Im{*a*_*m*_}
*y* < 0	0	− 4.297	0.000	0	4.983	0.000	0	118.056	0.000
	1	2.807	3.994	1	− 3.063	− 3.054	1	− 74.517	− 96.428
	2	1.566	− 0.364	2	− 1.347	− 0.264	2	− 40.421	3.934
	3	0.277	− 0.353	3	− 0.002	0.134	3	− 3.147	7.355
	4	0.102	0.097	4	− 0.169	− 0.006	4	− 3.964	− 2.101
	5	0.163	− 0.101	5	− 0.280	− 0.046	5	− 4.943	0.451
	6	0.120	− 0.072	6	− 0.056	− 0.112	6	− 2.236	1.211
	7	− 0.032	− 0.020	7	0.053	− 0.050	7	0.503	− 0.442
	8	0.076	− 0.014	8	− 0.122	0.012	8	− 1.429	− 1.425
	9	0.085	− 0.012	9	− 0.149	− 0.037	9	− 2.926	0.407
*y* < 0	0	− 4.705	0.000	0	0.000	0.000	0	122.438	0.000
	1	3.014	4.625	1	0.000	0.000	1	− 72.327	− 92.808
	2	1.899	− 0.495	2	0.000	0.000	2	− 41.436	1.203
	3	0.280	− 0.362	3	0.000	0.000	3	− 4.122	4.902
	4	0.084	0.052	4	0.000	0.000	4	− 2.619	− 3.729
	5	0.237	− 0.102	5	0.000	0.000	5	− 4.207	0.167
	6	0.084	− 0.093	6	0.000	0.000	6	− 2.591	1.917
	7	− 0.037	− 0.062	7	0.000	0.000	7	− 0.485	− 1.067
	8	0.081	0.028	8	0.000	0.000	8	− 1.947	− 2.582
	9	0.067	− 0.035	9	0.000	0.000	9	− 2.743	− 0.325
